# Dr. Kinglake's Observations on Hydrophobia

**Published:** 1812-10

**Authors:** Robert Kinglake

**Affiliations:** Taunton


*To the Editors of the Medical and Physical Journal.*

GENTLEMEN,

MUCH speculation has been held on the nature of Hydrophobia, as induced by the bite of a rabid animal. The various theories which have been proposed on the subject, have not satisfactorily elucidated the true character of the disease, nor have they suggested any mode of cure that might might be resorted to with any hopeful prospect of success. The disease is yet often occurring, and its destructive ravages are inflicted in defiance of all the resistance medical aid has been able to oppose to them. This insufficiency in the treatment of so direful a malady, evinces that the received opinion of the deadly quality and influence of its virulent cause, is too well founded. It is, however, unavailing, it is even worse, it is irksomely humiliating to medical science, to be often repeating the trial of inefficacious means. The common feelings of humanity, independently of that generous ardor usually prevailing to bring under control an untractable disease, is sufficient to warrant even the indulgence of conjecture in search of efficient modes of relief.

The prevention of this disease after the infliction of an infectious bite, has employed much device and contrivance; and it may be justly inferred, that the endeavor to benefit in this way has not been altogether fruitless. Much uncertainty indeed must always necessarily attach to all conclusions as to the efficacy of preventive means, from not knowing whether the animal that had inflicted a suspicious wound, was actually rabid, and whether the wounded person might have been susceptible of the diseased influence which the imparted virulence was calculated to produce. Positively, therefore, to decide in such questionable circumstances, would be taking more for granted than could be clearly proved, and would of course render any deductions from such premises in strictness inadmissible.

Excision of the bitten part, cauterizations, caustic applications, and an unwearied continuance of aqueous ablution, are means that should be severally and collectively employed, on the principle of making, if possible, *assurance double sure.* "*Venienti occurrite morbo*" should be, on these occasions, the actuating maxim, and its precept should be implicitly obeyed. Too much cannot be attempted for the important object of this practice; but, if all should fail, and the disease with its characteristic and hydra horrors should actually present, whither shall we fly for assistance? Do the archives of medicine record the instances in which this, or that, or any, mode of treatment has succeeded? Have not grim despair and agonizing death mocked every effort hitherto made to stem the progress, and prevent the mortal issue, of this virulent disease?

When, therefore, the hydrophobic malady has actually occurred, whether through the neglect or inefficacy of preventive means, it becomes the anxious object of medical treatment so far at least to repress its violence as to render its action, compatible with life, sufficiently long to afford an interval interval in which an attempt to relieve and cure may be subjected to trial. Forlorn indeed is that state that is, as it were, *ex rerum necessitate,* cut off from the possibility of being aided. In this irremediable view, however, have been regarded the nature and tendency of hydrophobic disease. Hence the justification for assailing this ruthless malady by any mode which the resources of medical speculation and experience may afford. *Anceps remedium melius est quam nullum*, is a rule of absolute authority in this case, and should be acted on with unyielding firmness.

Circumstanced then as the hydrophobic disease is with respect to its terrific features, and usual mortal termination, instead of offering useless condolence when it presents, and querulously lamenting the insufficiency of all known modes of relief, the utmost zeal should be employed in putting to the test of experiment such farther attempts as may be at all countenanced by either direct or analogical reasoning on the beneficial influence of any means that may be adopted.

In this experimental view of the treatment of hydrophobic disease, I have to suggest the strong resemblance that subsists between the real character of that affection and that of gastritis. In both, dissection has ascertained the appearance of inflammatory ailment. The stomachs of patients who have died of hydrophobia have exhibited to attentive examination strong traces of inflammatory disease. The œsophagus and tonsils have also partook in the stimulant effects of the inflamed stomach. This condition of stomach is so extremely irritable as to render the contact of even fluids insufferably painful. Contractile resistance, accompanied with urgent efforts to vomit, are induced by any attempt to swallow a fluid. The inverted action also of the muscular structure of the stomach and œsophagus is such, as to occasion all the horrors of ideal suffocation in any endeavor to overcome it. This I have had an opportunity of seeing in two cases of gastritis, to such a degree (particularly in one of the instances) as to afford a very striking resemblance to the characteristic features of the hydrophobic malady. In dissecting the gullet of persons who have died of hydrophobia, appearances have been observed very similar to those recited as obtaining in gastritis. Delirium is also common to the two diseases. The late Dr. Beddoes, but a short time before his death, had ascertained, by an adequate series of observations, that an inflamed aspect of the cardiac portion of the stomach was usually connected with a delirious affection of the brain, authorising the inference that these two conditions stood with each other in the relation of cause and effect. The highly sentient state of the stomach generally, and more particularly at its upper orifice, would account for its sympathetically involving in its morbid affections the nervous system at large, and especially the brain, as the grand source of sensibility.

It is clear then, from general reasoning as well as from the particular analogy obtaining between the apparent causes and symptoms of hydrophobia and gastritis, that the mode of attempting the cure of the one is also applicable to that of the other. It is obvious that gastritis can only be overcome by subduing, in the most effectual manner, the inflammatory action that constitutes the disease, and the mode of accomplishing this must chiefly consist in vascular depletion, carried to the utmost extent that may be compatible with life. Copious and frequent evacuations of blood are principally to be relied on. The abstraction of this fluid should be extended to at least sixteen ounces three times (at equal intervals) in the course of twenty-four hours, conjoined with vesication over the region of the stomach by way of counter-stimulus. The bowels should be kept lax by the frequent use of emollient clysters, and cold water should likewise be often injected into them, and also be frequently drank if swallowing be practicable.

This plan of treatment affords the best prospect of success in gastritis, and by parity of reason it would appear likely to prove more efficacious in hydrophobia than any other that could be adopted. The indication of cure in hydrophobia must consist in endeavoring to subdue the condition of active inflammation existing in the stomach, œsophagus, and fauces, which would be appropriately attempted by bleeding to the extent of twenty-four ounces, if deliquium should not be previously induced, repeating it at short intervals, as long as either firmness of arterial action or the symptoms of hydrophobic disease might remain. The region of the stomach, as in gastritis, may be also blistered as a counter-acting stimulus. The bowels should be kept freely open, and cold water may be frequently injected into them, whilst the same fluid may be as liberally taken by the mouth as the ability to swallow will permit. If the active disease should be thus overcome in the stomach and gullet, it is possible the patient might recover.

The deadly character of the disease would appear to depend on its having for its seat the stomach, which, of all the other vital organs, is that which is most likely to render every disease severely effecting it, dangerous and incurable. Life itself, and its various phenomena of health and disease, are so essentially connected with the sentient, irritable, sympathetic, sympathetic, and digestive, functions of the stomach, that the derangement of that viscus by inflammatory action, is fully capable of inducing, both directly and indirectly, a degree of disorder that must necessarily prove mortal. It is, perhaps, impossible to restrain the destructive violence of hydrophobic disease; it is possible that its intensity far exceeds any curative resistance which either nature or art could oppose to its ravages; this may be so, but such an irretrievable state of disease is not positively known to exist; it would be therefore gratuitous and quite irrational to rest a practical conclusion on imaginary grounds. It can only be put to the test of proof by trying the efficacy of probable means of relief; and, when every endeavor, founded either on the deductions of reason or the indication of experience, shall have failed, it may be justly regarded as an unattainable object until some new light be shed on the darkness that overshadows human knowledge on the subject.

In this view I have ventured to propose the depleting plan of treating hydrophobia, founded on the apparent resemblance subsisting between that disease and gastritis, knowing, from experience, that in the latter affection the treatment advised is the most efficacious that can be employed. It may be objected that the hydrophobic disease is induced by a specific action similar to what obtains in variola, morbilla, vaccina, syphilis, &c., and that lessening its violence, by reducing the generative powers of life by depletion, would be merely to diminish its force, and not to destroy the peculiarity of morbid condition, on which its nature depends. This may be true, yet it gains a truce, it keeps at bay a distemper that otherwise would speedily destroy life, and thus, either affords, as in other instances of specific disease, an opportunity for the disease to wear itself out by a gradual annihilation of farther susceptibility for the morbid stimulus, or admits of such aid from the resources of medical art as circumstances may suggest and require; so that, in whatever aspect the subject be considered, the endeavour to assist in the way proposed, would seem to be feasible, and is not without ground on which may be founded a rational expectation of benefit. To obtain relief and effect a cure in this truly tremendous malady, must be regarded as a medical desideratum of the first importance to afflicted humanity.

I am, &c.

ROBERT KINGLAKE.

